# Quantum Monte Carlo simulations of a giant {Ni_21_Gd_20_} cage with a *S* = 91 spin ground state

**DOI:** 10.1038/s41467-018-04547-4

**Published:** 2018-05-29

**Authors:** Wei-Peng Chen, Jared Singleton, Lei Qin, Agustín Camón, Larry Engelhardt, Fernando Luis, Richard E. P. Winpenny, Yan-Zhen Zheng

**Affiliations:** 1 Frontier Institute of Science and Technology (FIST), State Key Laboratory for Mechanical Behavior of Materials, MOE Key Laboratory for Nonequilibrium Synthesis and Modulation of Condensed Matter, and School of Science, Xi’an Jiaotong University, 99 Yanxiang Road, Xi’an, Shaanxi 710054 China; 2 Department of Physics and Astronomy, Francis Marion University, Florence, SC 29502 USA; 3 Instituto de Ciencia de Materiales de Aragón (ICMA) and Departamento de Física de la Materia Condensada, CSIC-Universidad de Zaragoza, E-50009 Zaragoza, Spain; 4 Department of Chemistry and Photon Science Institute, The University of Manchester, Manchester, M13 9PL UK

## Abstract

The detailed analysis of magnetic interactions in a giant molecule is difficult both because the synthesis of such compounds is challenging and the number of energy levels increases exponentially with the magnitude and number of spins. Here, we isolated a {Ni_21_Gd_20_} nanocage with a large number of energy levels (≈5 × 10^30^) and used quantum Monte Carlo (QMC) simulations to perform a detailed analysis of magnetic interactions. Based on magnetization measurements above 2 K, the QMC simulations predicted very weak ferromagnetic interactions that would give a record *S* = 91 spin ground state. Low-temperature measurements confirm the spin ground state but suggest a more complex picture due to the single ion anisotropy; this has also been modeled using the QMC approach. The high spin and large number of low-lying states lead to a large low-field magnetic entropy (14.1 J kg^−1^ K^−1^ for Δ*H* = 1 T at 1.1 K) for this material.

## Abstract (web summary)

Paramagnetic metal clusters with large ground spin states often possess attractive magnetic behaviors for information storage or solid-state cooling applications. Here, the authors design a giant {Ni_21_Gd_20_} cage, which using quantum monte carlo simulations they predict to possess a spin ground state approaching *S* = 91.

## Introduction

Paramagnetic metal clusters possessing large ground spin state (*S*) often show attractive magnetic behavior with potential applications such as single-molecule magnets (SMMs)^1,2^, magnetic refrigerants^3,4^, and contrast agents for magnetic resonance imaging (MRI)^5,6^. To date, the highest ground spin states reported are *S* = ^83^/_2_ for a {Mn_19_} cage^7^, *S* = ^61^/_2_ for a {Mn_49_} cage^2^, *S* = 45 for an {Fe_42_} cage^8^, and *S* = 60 for a {Fe_10_Gd_10_} nano-torus^9^. The accurate magnetic analysis of such giant species is challenging because the number of energy levels increases exponentially with the magnitude and number of spins. For example, the famous SMM, {Mn_12_} requires a diagonalization of a matrix with the dimension of 10^8^ and this has only recently been achieved^10–12^. Quantum Monte Carlo (QMC) simulation may provide a useful alternative for the investigation of large systems^13^. Successful examples include both ring^14–17^ and cage^18^ structures. A further level of complexity can arise if significant single-ion anisotropy is present as this can compete with exchange interactions in deciding low-temperature physics.

Application of molecules with very high spin as magnetic refrigerants^19–21^, requires molecules with a large spin ground state, weak ferromagnetic exchange, and negligible magnetic anisotropy^3,4^. Cages based on gadolinium ions are excellent candidates to achieve such goals^22–24^ if ferromagnetic exchange can be induced. Controlling the sign of magnetic exchange is difficult, but we noticed two cases where ferromagnetic exchange is normally observed. Firstly, the *syn*–*anti* bridging mode in nickel(II) carboxylate complexes usually causes ferromagnetic exchange between nickel(II) ions^25^. Secondly, where two gadolinium(III) ions are bridged by a single O-atom, a large Gd–O–Gd angle (>110.9°) yields ferromagnetic exchange between Gd ions^26^. The {Ni_21_Ln_20_} cages (Ln = Pr and Nd) reported by Kong et al.^27^ contain the correct bridging mode for the Ni(II) units, and the Ln–O–Ln angles are large. However, this group was unable to produce the Gd analog, and the yields reported of other {Ni_21_Ln_20_} clusters were low. These compounds feature 2-hydroxyl-acetate as a ligand, and this is formed in situ from the decomposition of iminodiacetic acid (IDAH_2_), which was the ligand added to the reaction. This in situ formation of a key ingredient probably explains the low yields.

Therefore we carried out similar chemistry, but included diphenylglycolic acid (DPGAH_2_), which has the same donor groups as 2-hydroxyl-acetate. This produces the spherical cage complex [Ni_21_Gd_20_(OH)_24_(IDA)_21_(DPGA)_6_(C_2_O_4_)_3_(NO_3_)_6_(CH_3_COO)_3_(H_2_O)_12_]·Br_5_·(NO_3_)_4_·20CH_3_OH·30H_2_O (**1**) in good yield. QMC simulations were carried out to analyze the magnetic exchange in this nanocage {Ni_21_Gd_20_}. The magnetic metal centres are ferromagnetically coupled, approaching a *S* = 91 spin ground state, but single-ion anisotropy competes with the exchange interactions, leading to a more complex magnetic state with large low field entropy (14.1 J kg^–1^ K^–1^ for Δ*H* = 1 T at 1.1 K), which is a new record in cryogenic cluster-based magnetic refrigerants to the best of our knowledge^19–21^.

## Results

### Structure of {Ni_21_Gd_20_} cage

**Fig. 1:**
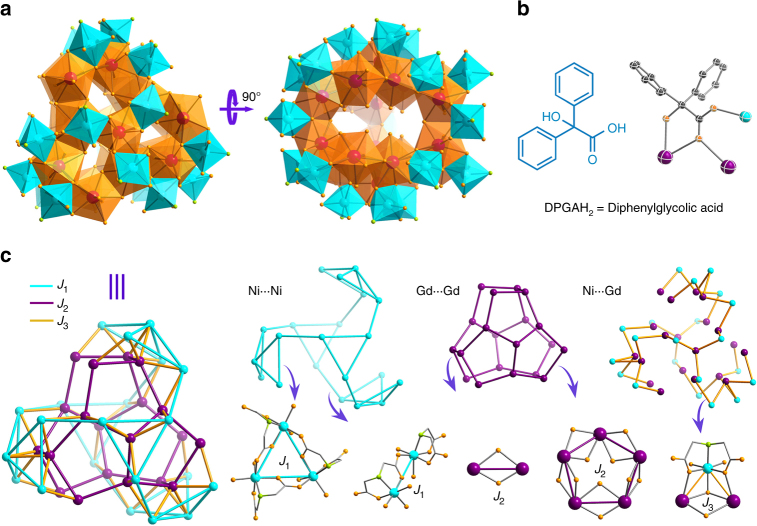
The structure of nanocage {Ni_21_Gd_20_} determined by X-ray crystallography. **a** The polyhedron structures of {Ni_21_Gd_20_} core with the organic ligands removed for clarity; **b** The coordination mode of the DPGA^2–^ ligand in this compound; **c** The magnetic coupling schemes of the metal centers in {Ni_21_Gd_20_} core. *J*_1_ = Ni∙∙∙Ni, *J*_2_ = Gd∙∙∙Gd, *J*_3_ = Ni∙∙∙Gd; Color codes: Gd purple, Ni cyan, N green, O orange, C gray

Single-crystal X-ray diffraction analyses reveal that **1** crystallizes in the hexagonal space group *P*6_3_*/m* (Supplementary Table 1). The spherical structure of the cationic cluster may be viewed as constructed from an inner {Gd_20_} core and the outer {Ni_21_} shell (Fig. 1a and Supplementary Fig. 1). The inner {Gd_20_} core contains two bowl-like Gd_10_ subunits made up of a triangle of three pentagonal Gd_5_(NO_3_) fragments that share four Gd(III) ions (Supplementary Fig. 1). The NO_3_^−^ ions sit in the middle of the pentagonal Gd_5_(NO_3_) fragments with a 5.222 bridging mode (Harris notation). Three *µ*−OH^−^, three *μ*-acetate bridges and three C_2_O_4_^2–^ ligands link the two bowl-like Gd_10_ subunits to form the {Gd_20_} core. The Gd–O distances (2.29–2.72 Å) and the Gd(III)∙∙∙Gd(III) separations (3.89–4.18 Å) in this core are comparable to other reported Gd-based clusters. Note that the Gd–O–Gd angles ranging from 104.7° to 120.3° average at 111.0° (Supplementary Table 2), which usually causes ferromagnetic exchange between Gd(III) ions^26^.

The inner {Gd_20_} core and outer {Ni_21_} shell are connected by six DPGA^2–^ ligands, 24 *µ*_3_-hydroxides and twenty-one O atoms of IDA^2–^ to form a spherical framework {Ni_21_Gd_20_} with the Ni(II)∙∙∙Gd(III) distances ranging from 3.46 to 3.63 Å. In the external {Ni_21_} shell (Supplementary Fig. 1), there are three {Ni(IDA)}_5_ fragments and two {Ni(IDA)}_3_ units, which are bound to carboxylate groups from IDA ligands. The butterfly-shaped {Ni(IDA)}_5_ fragments are located in the equatorial zones of the sphere, while two {Ni(IDA)}_3_ units sit at the two poles. The IDA ligands adopt the *syn–anti* bridging mode to connect adjacent Ni(II) ions to form the {Ni(IDA)}_21_ shell. The Ni–O bond distances, Ni–N bond distances and Ni(II)∙∙∙Ni(II) separations range from 1.99 to 2.10, 2.08 to 2.11, and 5.16 to 5.28 Å (Supplementary Table 2), respectively.

### Magnetic properties

**Fig. 2:**
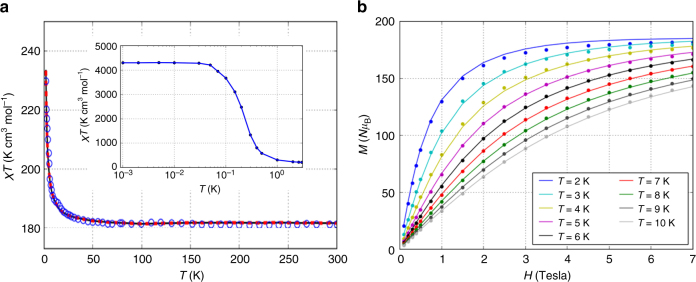
The magnetic characteristics for compound 1. **a** The temperature dependences of *χT* versus *T*. The experimental data are shown as circles, and the solid curve is for the single *J*-model, assuming *D*_Ni_ = 0 with *J*/*k*_B_ = –0.033 K (best fit). **b** Magnetization versus field for several fixed values of temperature using the single *J*-model (with *D*_Ni_ = 0) that provides the best fit to the susceptibility. The experimental data are shown as dots and theory data shown as solid curve. (Inset of **a**) Low-temperature predictions of the isotropic Heisenberg model for *χT* versus *T*

The magnetic behavior of a polycrystalline powder sample of **1** was studied in the temperature range *T* = 2–300 K under a 1000 Oe dc field, as shown in Fig. 2a. The *χT* value of 181.3 K cm^3^ mol^–1^ at room temperature is only slightly below the high-temperature Curie limit of 182.9 K cm^3^ mol^–1^ that would be expected for 21 uncorrelated Ni(II) ions (*S* = 1, *g* = 2.2) and 20 uncorrelated Gd (III) ions (*S* = 7/2, *g* = 2). Upon cooling, *χT* remains relatively constant until 35 K and then increases abruptly, reaching a value of 229.8 K cm^3^ mol^–1^ at 2 K without saturation (Fig. 2a). This rapid rise of *χT* suggests that predominantly ferromagnetic interactions are present. This is supported by fitting the inverse molar susceptibility between *T* = 100 and 300 K using the Curie–Weiss equation, which gives *C* = 180.8 K cm^3^ mol^–1^ and *θ* = 0.36 K (Supplementary Fig. 2).

The field-dependent molar magnetization, *M*(*H*), of **1** was also measured for the temperature range *T* = 2–10 K in the field range *H* = 0–7 T (Fig. 2b). Fig. 2b shows that for *T* = 2.0 K, *M*(*H*) rapidly increases for *H* < 1 T, and then begins to flatten out for *H* > 2 T, nearly saturating and reaching a value of *M* = 181.7 *Nμ*_B_ (where *N* is the Avogadro constant and *μ*_B_ is the Bohr magneton). This value is very close to the expected high-field limit of *M* *=* 182 *Nμ*_B_ for *g* = 2.0 and *S* = 91. The measured magnetization curves are slightly higher than the corresponding Brillouin curves corresponding to 41 non-interacting spins, and very substantially lower than the Brillouin curve for *S* = 91 ground spin state (Supplementary Fig. 3). This is again consistent with weak ferromagnetic coupling within the molecule.

The detailed investigation of inter-ion exchange coupling in giant 3d–4f polymetallic complex systems has always been a challenge due to the large number of magnetic states. Since **1** consists of 20 Gd(III) ions with *S* = 7/2 and 21 Ni(II) ions with *S* = 1, the number of the states is ≈ 5 × 10^30^, so matrix diagonalization is not possible. Instead, we calculated *χT* using a QMC method with the stochastic series expansion implementation from Algorithms and Libraries for Physics Simulations (ALPS), as shown in Fig. 2 (solid curves)^28,29,30^. For these simulations, we assumed a Hamiltonian of the form

$$\hat H = 	J_1\mathop {\sum }\limits_{\begin{array}{*{20}{c}} {\mathrm {Ni}},{\mathrm {Ni}} \\ {i,j} \end{array}} \hat s_i \cdot \hat s_j + J_2\mathop {\sum } \limits_{\begin{array}{*{20}{c}} {{\mathrm {Gd}},{\mathrm {Gd}}} \\ {i,j} \end{array}} \hat s_i \cdot \hat s_j + J_3\mathop {\sum }\limits_{\begin{array}{*{20}{c}} {{\mathrm {Ni}},{\mathrm {Gd}}} \\ {i,j} \end{array}} \hat s_i \cdot \hat s_j \\ 	 + D_{{\mathrm{Ni}}}\mathop {\sum }\limits_{\begin{array}{*{20}{c}} {\mathrm {Ni}} \\ j \end{array}} \hat s_{jz}^2 - \mu _{\mathrm {B}}H\mathop {\sum }\limits_i g_i\hat s_{iz}$$

which uses the sign convention where *J* < 0 represents a ferromagnetic interaction and omitting the single ion magnetic anisotropy on the Gd(III) sites.

The Ni(II)∙∙∙Ni(II), Gd(III)∙∙∙Gd(III), and Ni(II)∙∙∙Gd(III) distances in this cluster range from 5.16 to 5.28, 3.89 to 4.18, and 3.46 to 3.63 Å, respectively; so three exchange interactions were used to describe the system: *J*_1_ corresponds to the Ni∙∙∙Ni interactions, *J*_2_ to Gd∙∙∙Gd, and *J*_3_ to Ni∙∙∙Gd (Fig. 1c). In order to reduce the size of the parameter space to be explored, we initially set *D*_Ni_ = 0, which corresponds to a purely isotropic Heisenberg Hamiltonian with three exchange constants. The values *g*_Gd_ = 1.99 and *g*_Ni_ = 2.196 were determined from the measured susceptibility, especially the high-temperature data.

Using the isotropic Heisenberg Hamiltonian described above, we were able to get a very good fit to the measured data in the range *T* = 2–300 K, as shown in Fig. 2. When allowing all three exchange interactions to vary, the parameter values that gave the best fit were: *J*_1_/*k*_B_ = –0.0305 K, *J*_2_/*k*_B_ = –0.0302 K, and *J*_3_/*k*_B_ = –0.0516 K (all ferromagnetic interactions). Since these three *J* values are very similar, we considered a single-*J* model, constraining all three *J* values to be equal to each other. For the single-*J* model, we found *J*/*k*_B_ = –0.033 K, and the goodness of fit for the single *J* value was virtually identical to the goodness of fit for the three-*J* model. (See Supplementary Figs. 4 and 5 for details regarding the goodness of fit.)

The ferromagnetic exchange interactions described above predict a *S* = 91 spin ground state with *χT* ≈ 4300 K cm^3^ mol^–1^ at very low temperatures, as shown in the inset of Fig. 2a. However, this spin ground state is not isolated, and to this point we have neglected any single-ion anisotropy by setting *D*_Ni_ = 0, which could be important for Ni(II). It is perhaps surprising that an isotropic model, which we have used to this point, models the 2–300 K data so well.

**Fig. 3:**
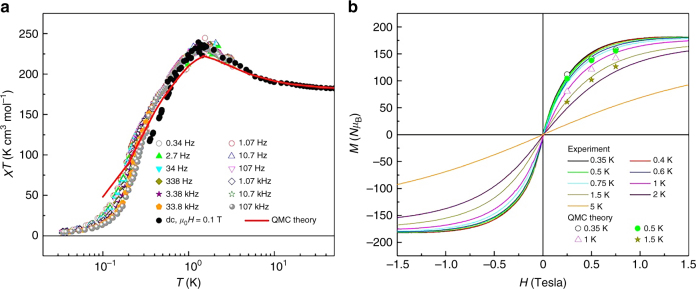
Very low-temperature magnetic behavior. **a** Temperature dependence of *χT* measured at *H* = 0 and in the region of very low temperatures for different frequencies. Dc data measured under a magnetic field *H* = 0.1 T are also shown. Experimental data are represented with symbols, and the solid line represents the best-fit for the anisotropic model described in the text. **b** Magnetization versus field isotherms measured at very low temperatures. Solid curves: measured magnetizations at temperatures given. Symbols: QMC calculated magnetizations using the best-fit parameters given in the text

To establish whether or not this prediction of a record *S* = 91 spin ground state is correct, we carried out lower temperature magnetic measurements (supplementary Fig. 6). These results show that the picture of an isotropic *S* = 91 spin ground state is simplistic. The zero-field ac susceptibility (supplementary Figs. 7 and 8) departs, below 2 K, from the paramagnetic behavior observed at higher temperatures. The *χT* product (Fig. 3a) reaches a maximum at about 1.5 K and then decreases. Also, *χ* becomes frequency-dependent below 350 mK, which shows the existence of slow magnetic relaxation processes. The characteristic spin-lattice relaxation time increases with decreasing *T* and becomes of the order of seconds near 0.1 K (supplementary Fig. 9). This slow magnetic relaxation can be associated with a zero-field splitting that originates from the finite magnetic anisotropy of the constituent Gd(III) or Ni(II) ions. The activation energy *U*/*k*_B_ = 3.3 K that governs these processes is close to values found for Gd-based single-ion magnets^31^.

To model these new data, we expanded our QMC approach to include an axial zero-field splitting for the Ni(II) ions, *D*_Ni_ ≠ 0. We initially used the single *J*-model with *D*_Ni_ ≠ 0, which gave two free parameters, *J* and *D*_Ni_; but within this two-dimensional parameter space it was not possible to produce a peak in *χT* around *T* = 1.5 K. Thus we expanded our search to the full four-dimensional parameter space of the Hamiltonian (*J*_1_, *J*_2_, *J*_3_, *D*_Ni_). There is now a danger of over-parameterization, however a key feature of the QMC approach is that we are able to examine a massive segment of parameter space, and hence the possibility of false minima is reduced. In this expanded parameter space, we were indeed able to get a reasonably good fit to the measured *χT* curve, as shown in Fig. 3a. The set of parameters that gives this best fit is: *J*_1_/*k*_B_ = –0.0225 K, *J*_2_/*k*_B_ = –0.0113 K, and *J*_3_/*k*_B_ = –0.225 K (all ferromagnetic) and *D*_Ni_/*k*_B_ = 2.5 K (hard axis anisotropy), where—although these *J* values are listed using three digits—we estimate that a range of about ±10% around each parameter value will still give a similarly good fit. This model was also used to compute the *M* versus *H* data shown as filled circles in Fig. 3b. For each of these low temperatures, we find that this model gives magnetization values that are slightly higher than the measured values; but for *T* ≥ 2 K, this anisotropic model actually agrees with the measured magnetization slightly better than the isotropic model. (See Supplementary Fig. 10, as compared with Fig. 2.)

The magnetic entropy changes ∆*S*_m_ were evaluated by applying the Maxwell relation –∆*S*_m_(*T*) = ∫[*∂M*(*T,H*)/*∂T*]_*H*_d*H* on the cluster **1**. Calculated entropy changes obtained from magnetization and heat capacity (Supplementary Fig. 11) data give a maximum value of 34.8 J kg^–1^ K^–1^ at 3 K and 7 T for **1** (Supplementary Fig. 12), which is comparable to other Gd-based polymetallic clusters (Supplementary Table 3)^2^, but smaller than the maximum entropy value judged by the function of –∆*S*_m_ = n*R*ln(2 *S* + 1) = 64.7 *R*, which corresponds to 45.8 J kg^–1^ K^–1^ for **1**. The coupling between the Gd(III) and Ni(II) ions, as well as their finite magnetic anisotropies, reduce the maximum achievable entropy content because these interactions partly lift the level degeneracy at *H* = 0. However, the dominant ferromagnetic couplings also enable reaching sizeable –Δ*S*_m_ values at moderate magnetic fields. A large low-field magnetic entropy change, reaching 14.1 J kg^–1^ K^–1^ for a magnetic field change of 1 T at 1.1 K, is observed (Table 1).

**Table 1:** ∆*S*_M_ at low-field (<2 T) for reported polymetallic molecules

Complexes	–∆*S*_M_ (J kg^–1^ K^–1^) 2.0 T	–∆*S*_M_ (J kg^–1^ K^–1^) 1.0 T	–∆*S*_M_ (J kg^–1^ K^–1^) 0.5 T	*T* (K)	Ref
{Gd^III^_24_}	22.6	10	6	2.0	32
{Ni^II^_21_Gd^III^_20_} (**1**)	19.8	14.1	6.6	2.0/1.1	This work
{Ni^II^_64_Gd^III^_96_}	17	7.0	2.0	3.0	22
{Gd^III^_10_}	17	8.0	3.5	2.0	33
{Co^II^_4_Gd^III^_10_}	15	3.7	2.3	3.0	34
{Co^II^_9_Co^III^Gd^III^_42_}	14	5.0	2.0	2.0	35
{Gd_12_Mo_4_}	14	5.0	1.8	3.0	36
{Gd^III^_48_}	13.7	5.0	2.0	1.8	37
{Ni^II^_10_Gd^III^_42_}	13.5	4.8	2.0	2.0	35
{Mn^II^_4_Gd^III^_6_}	13.5	5.0	1.5	2.0	38

## Discussion

The analysis of the magnetic properties of giant heterometallic paramagnetic molecules, such as the one presented here, is rare due to the multiple magnetic couplings and the large number of magnetic states. In **1**, there are three different inter-ion exchange interactions and 99 connections (30 *J*_1_, 27 *J*_2_ and 42 *J*_3_, see Fig. 1c), and the number of the states reaches 5 × 10^30^ even before we allow for any anisotropy. Thus the simulation of the magnetic interaction in such a large system through traditional fitting methods is impossible. QMC simulations were performed which allow us to model the magnetic behavior from 0.35 to 300 K using three exchange interactions and single-ion anisotropy for nickel. The model predicts weak ferromagnetic exchange interactions, consistent with previous observations^22,25–27,39–41^, and a spin ground state *S* = 91 and a very large low field magnetic entropy change (14.1 J kg^–1^ K^–1^ for Δ*H* = 1 T at 1.1 K).

## Methods

### Materials and measurements

All reagents and solvents for the syntheses were purchased from commercial sources and used as received. Elemental analyses (C, H, and N) were performed on a Vario EL III elemental analyzer. Infrared spectra (4000−400 cm^−1^) of all samples were recorded on a Thermo Scientific Nicolet 6700 FT-IR spectrophotometer. Magnetic susceptibility measurements were performed on a Quantum Design MPMS-XL7 SQUID magnetometer operating between 2 and 300 K and dc-applied fields ranging from 0 to 7 T. The powder sample was fixed with eicosane and placed in a calibrated gelatine capsule, which was held at the centre of a straw. To prevent the loss of the crystal solvent in **1**, the straw was immediately transferred into the sample chamber of the SQUID at 100 K under helium atmosphere. After the sample space was vent with helium gas and the measurement starts. Background subtractions were performed for the sample holder. Diamagnetic corrections for the sample were applied using with Pascal constants.

Ac susceptibility measurements were extended to the region of very low temperatures by using a home-made micro-SQUID susceptometer^42^. The micro-SQUID has a gradiometric design, with two 30 μm wide Nb loops that act as the pick-up coils of the susceptometer. Each of these two loops is placed inside a solenoid that generates the excitation ac magnetic field. The device works in the frequency range 0.01 Hz–250 kHz. It is installed inside the mixing chamber of a ^3^He-^4^He dilution refrigerator, which enables performing experiments between 13 mK and 4.2 K. A grain of powder sample was embedded in grease and placed on top of one of the SQUID loops. The amplitude of the ac magnetic field was 0.05 Oe. Magnetization isotherms were measured between 0.35 K and 5 K with a home-made micro-Hall magnetometer. The sample was placed on the edge of one of the three Hall crosses. A magnetic field *H* < 2 T was applied along the plane of the sensor to minimize its intrinsic bare signal. This signal was calibrated and then subtracted from the results. Heat capacity data were measured, down to *T* = 0.35 K, with a commercial physical property measurement system that makes use of the relaxation method^43^.

### Synthesis of {Ni_21_Gd_20_(OH)_24_(IDA)_21_(DPGA)_6_(C_2_O_4_)_3_(NO_3_)_6_(CH_3_COO)_3_(H_2_O)_12_}·Br_5_·(NO_3_)_4_·30H_2_O·20CH_3_OH (**1**)

Iminodiacetic acid (0.133 g, 1.0 mmol), diphenylglycolic acid (0.228 g, 1.0 mmol), Ni(CH_3_COO)_2_·4H_2_O (0.311 g, 1.25 mmol), Gd(NO_3_)_3_·6H_2_O (0.564 g, 1.25 mmol), KBr (0.1 g, 1.7 mmol) and triethylamine (3.0 mmol) were dissolved in mixed solvent of H_2_O/CH_3_OH (8 mL, v/v = 1:1). This solution was then sealed in a 15 mL Teflon-lined stainless steel vessel and heated at 160 °C for 3 days. At a rate of 5 °C/h, the system was allowed to cool to room temperature. Green block-shaped crystals of **1** were collected, washed thoroughly with methanol, and dried in air at room temperature (yield ca. 35% on the basis of Ni). Elemental analysis calcd (%) for C_200_H_362_N_31_Ni_21_Br_5_Gd_20_O_236_: C 20.44, H 3.10, N 3.69; Found: C20.22, H 2.97, N 3.60. Infra-red (KBr disc): ñ = 3434 (m), 1573 (s), 1412 (s), 1108(w), 1020(m), 954 (w), 727 (m), 670 (m), 615 (m).

### X-ray structure determination

Single-crystal X-ray diffraction data were collected on a Bruker Apex DUO diffractometer with Mo Kα radiation (*λ* = 0.71073 Å) at 296 K. The structure was solved by direct methods and all non-H atoms were subjected to anisotropic refinement by full-matrix least-squares refinement on *F*^*2*^ using SHELXTL. Because of disorder, the two phenyl rings of DPGA^2−^ were both splitted into two parts (C19-C24 and C19A-C24A for one phenyl ring, and C25-C30 and C25A-C30A for another one), which were refined under AFIX 66. There are 20 disordered methanol molecules and 30 disordered water molecules per formula unit that were removed by SQUEEZE in the refinement, but accurately confirmed by both elemental analyses and charge balance. Refinement parameters and crystallographic data for **1** are shown in Supplementary Table 1.

### Data availability

The X-ray crystallographic coordinates for structure reported in this study have been deposited at the Cambridge Crystallographic Data Centre (CCDC), under deposition number 1501498. The data can be obtained free of charge via www.ccdc.cam.ac.uk/data_request/cif. All other data are available from the authors upon reasonable request.

## Electronic supplementary material

Supplementary Information
