# Supplementary material for: Quantum Monte Carlo simulations of a giant {Ni21Gd20} cage with a S = 91 spin ground state
Source: Nat Commun. 2018 May 29;9:2107. doi: 10.1038/s41467-018-04547-4 (PMC5974011; doi:10.1038/s41467-018-04547-4)
Supplement: Supplementary file 1 — Supplementary Information [file 41467_2018_4547_MOESM1_ESM.pdf]

*Supplementary Information:*

# Quantum Monte Carlo Simulations of Magnetic Coupling in a Giant $\{Ni_{21}Gd_{20}\}$ Cage with a Record $S$ = 91 Spin Ground State

Wei-Peng Chen, Jared Singleton, Lei Qin, Agustín Camón, Larry Engelhardt, Fernando Luis, Richard E. P. Winpenny and Yan-Zhen Zheng

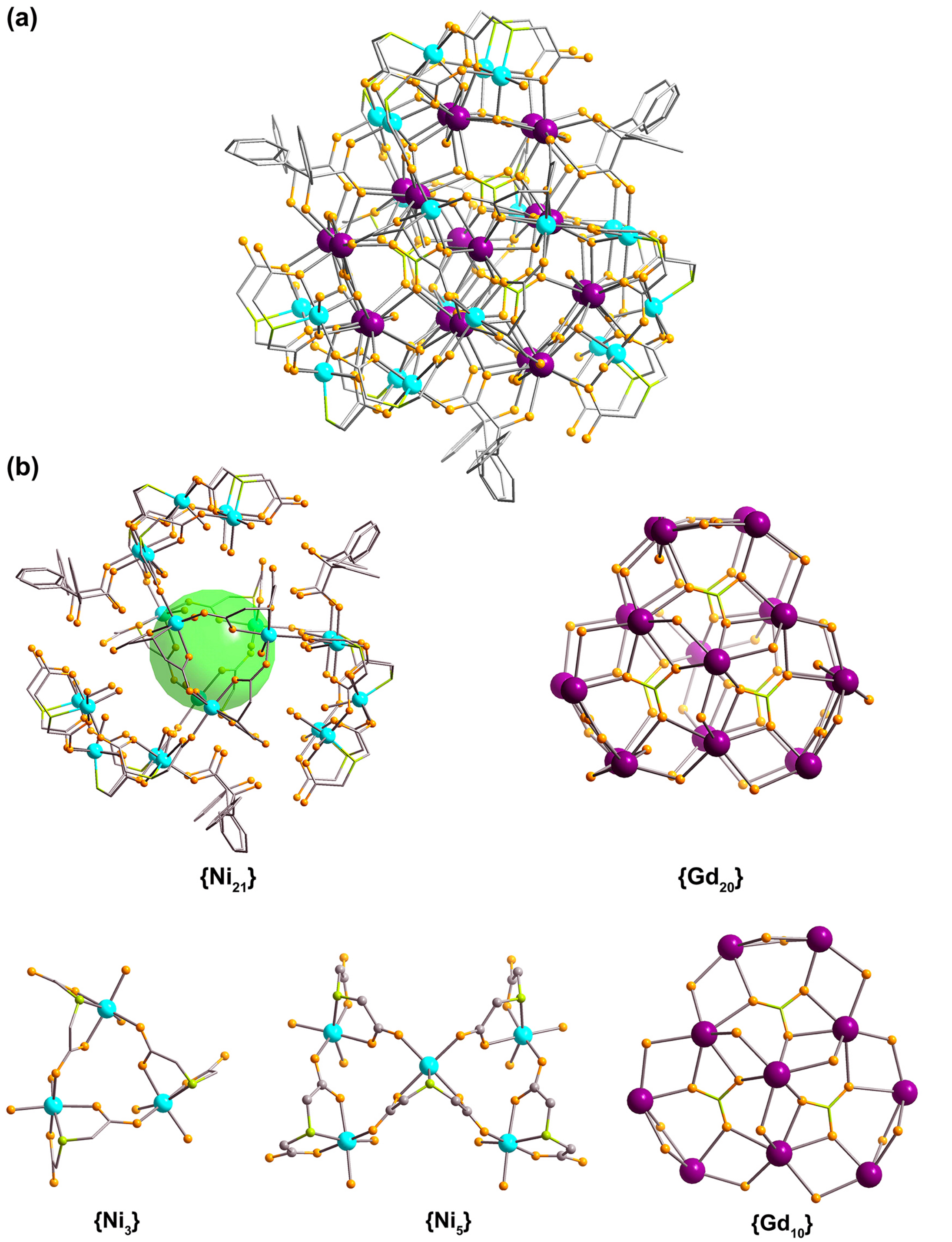


**Supplementary Figure 1** (a) The ball and stick view for the cationic dual shell cluster $\{Ni_{21}Gd_{20}\}$; (b) The structure details for the external $\{Ni_{21}\}$ shell and inner $\{Gd_{20}\}$ core; Gd purple, Ni cyan, N green, O orange, C gray.

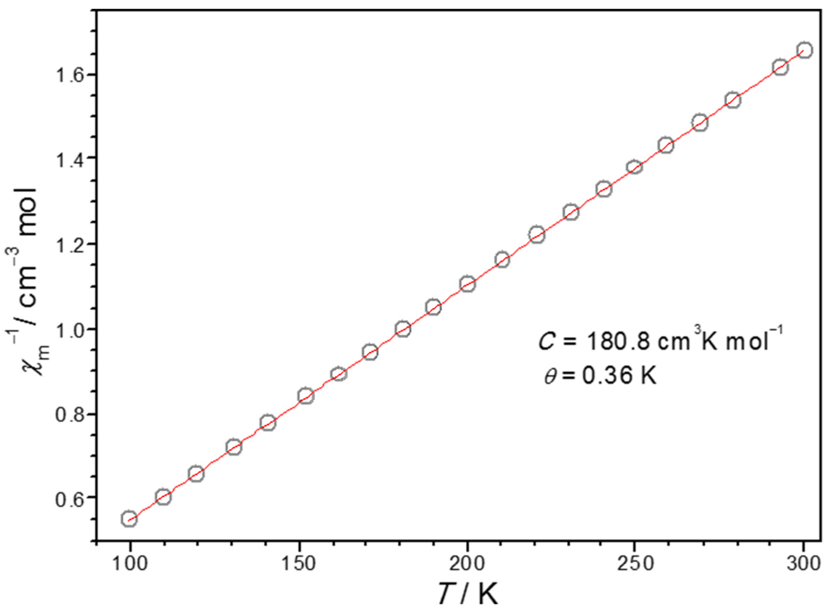


**Supplementary Figure 2** The plot of $\chi_M^{-1}$ *vs.* *T* for compound **1**.

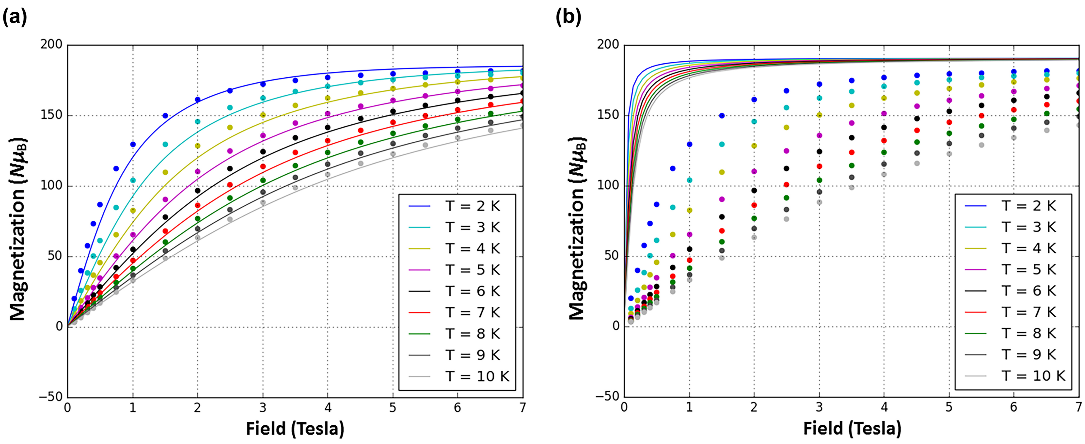


**Supplementary Figure 3** Magnetization versus field for several fixed values of temperature, with experimental data shown as circles and theory data shown as solid curves. (Left) Assumes zero coupling between sites, computed as a sum of Brillouin functions for 41 non-interacting spins ($S$ = 7/2 and $g$ = 1.99 for Gd(III) ions; $S$ = 1 and $g$ = 2.196 for Ni(II) ions). (Right) Assumes strong coupling, treating the system as a single spin with $S$ = 91 with $g$ = 2.093 (average).

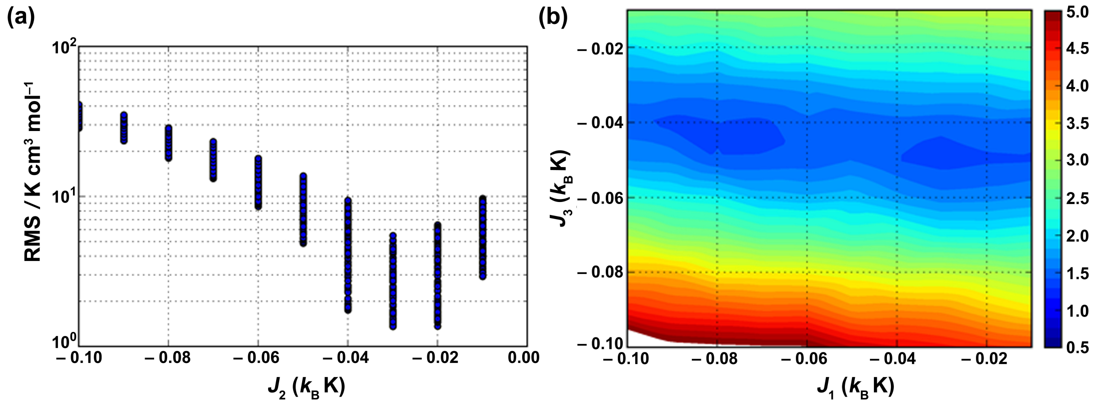


**Supplementary Figure 4** RMS deviation between model and experiment demonstrating the dependence of the goodness of fit on the parameters $J_1$, $J_2$, and $J_3$, assuming $D_{Ni} = 0$. (a) RMS deviation versus $J_2$, showing a clear minimum around $J_2/k_B = -0.03$ K. (b) Contour plot showing the RMS deviation as a function of both $J_1$ and $J_3$ for a fixed value of $J_2/k_B = -0.03$ K. The colors are defined in the color bar using units of K cm$^3$ mol$^{-1}$. There is a narrow minimum around $J_3/k_B \approx -0.04$ K, whereas $J_1$ has a very broad minimum.

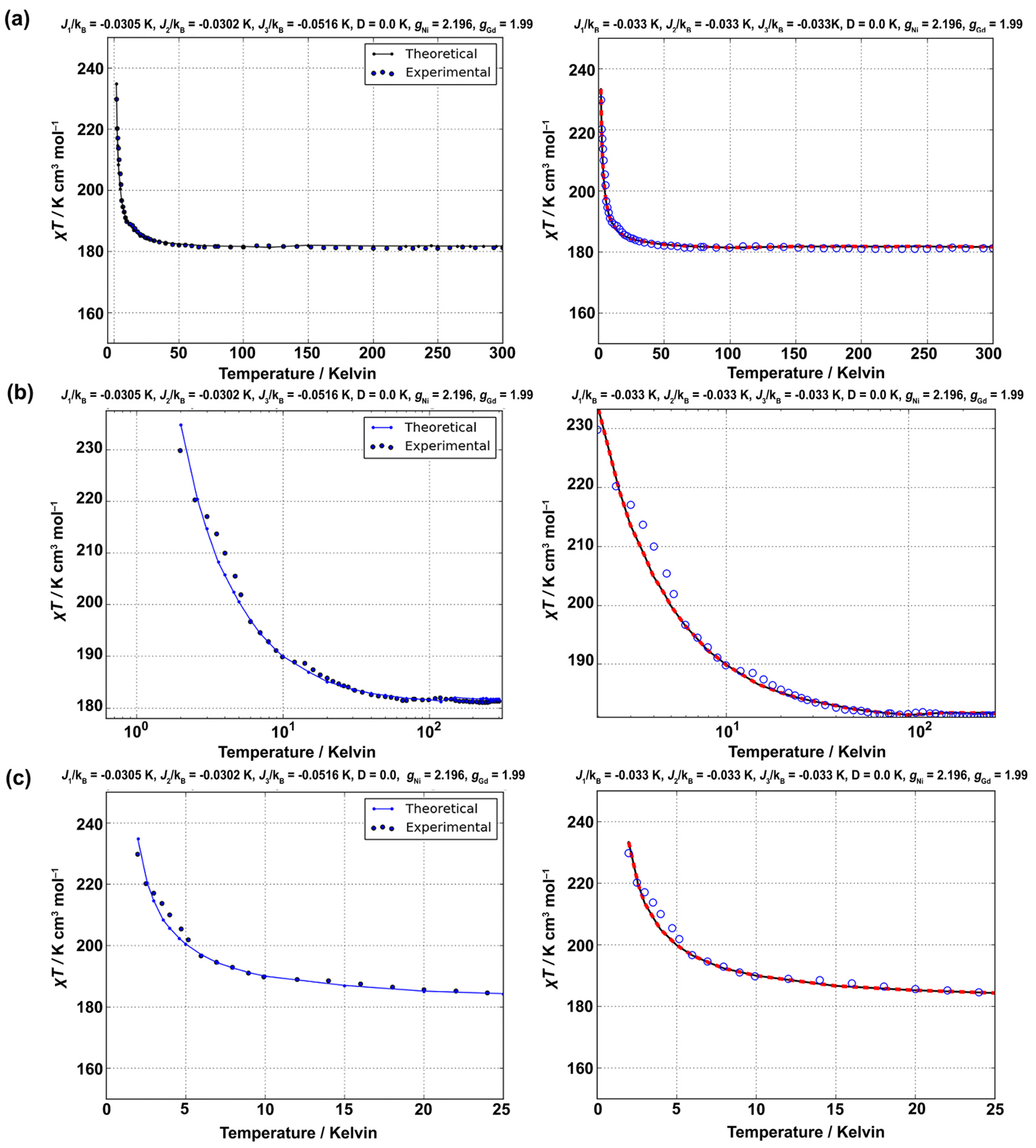


**Supplementary Figure 5** Experiment (dots) and theory (solid curves) for the set of parameter values that gave the best fit when assuming $D_{\mathrm{Ni}} = 0$. (Left: 3 *J*-Model, where all three *J* values are allowed to vary. Right: single *J*-Model, where all three *J* values to be equal). The plots (a) for full temperature range in linear scale, (b) for full temperature range in log scale, and (c) for low-temperature measurements.

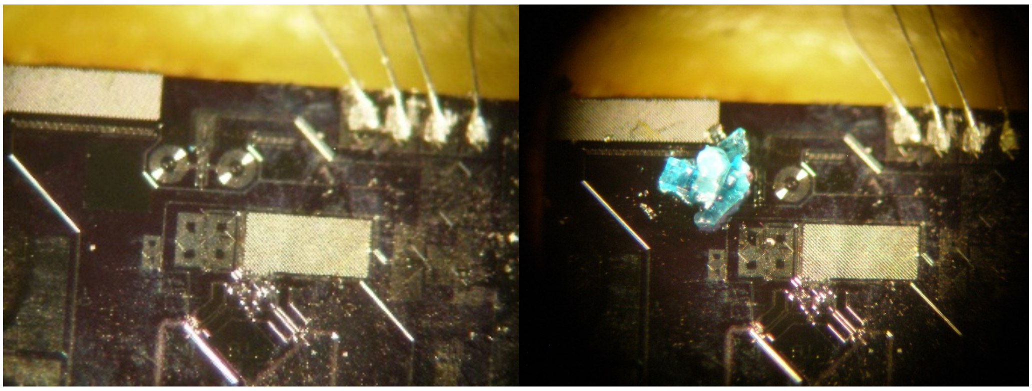

**Supplementary Figure 6** Left, microscopy image of the empty micro-SQUID ac susceptometer. Right, image of a polycrystalline sample of **1** placed on top of one of the micro-SQUID loops.

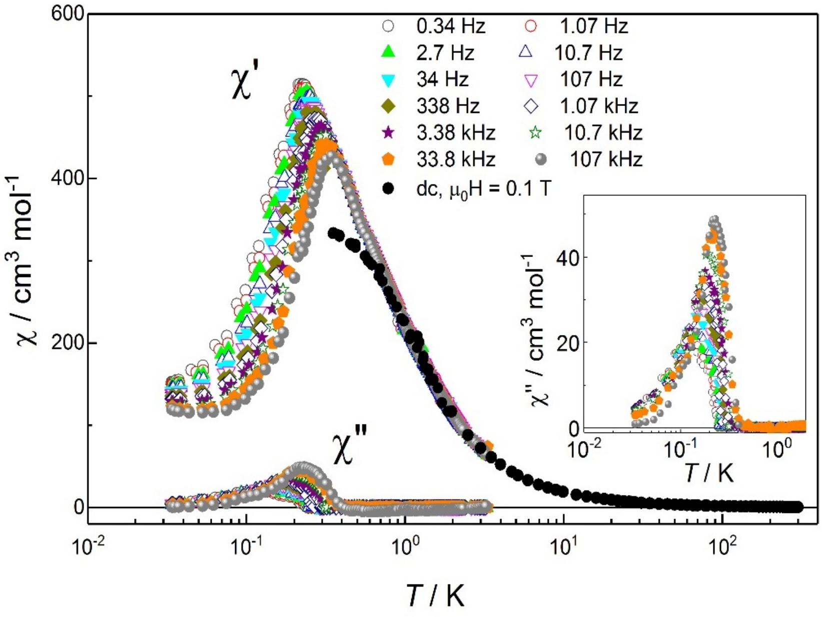


**Supplementary Figure 7** In-phase χ' and out-of-phase χ'' components of the ac magnetic susceptibility of **1** measured at $H = 0$ and for different frequencies with a micro-SQUID susceptometer. Dc susceptibility data measured at $\mu_0 H = 0.1$ T are also shown. The inset shows an enlarged view of the out-of-phase susceptibility data.

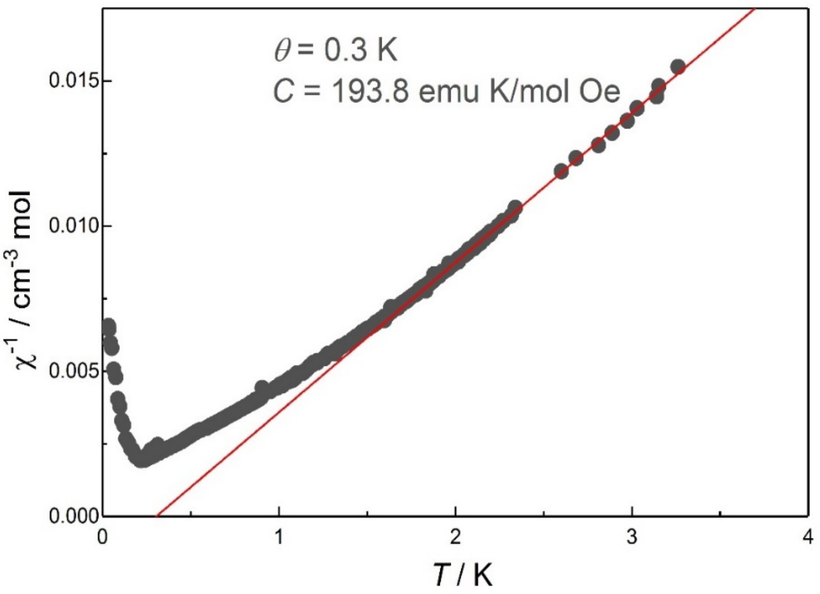


**Supplementary Figure 8** Reciprocal magnetic susceptibility of **1** measured at $H = 0$ and 0.338 Hz in the very low $T$ region. Data measured above $T = 0.22$ K correspond to the thermal equilibrium response. The solid line is a least squares fit of a Curie-Weiss law to data measured above 1 K.

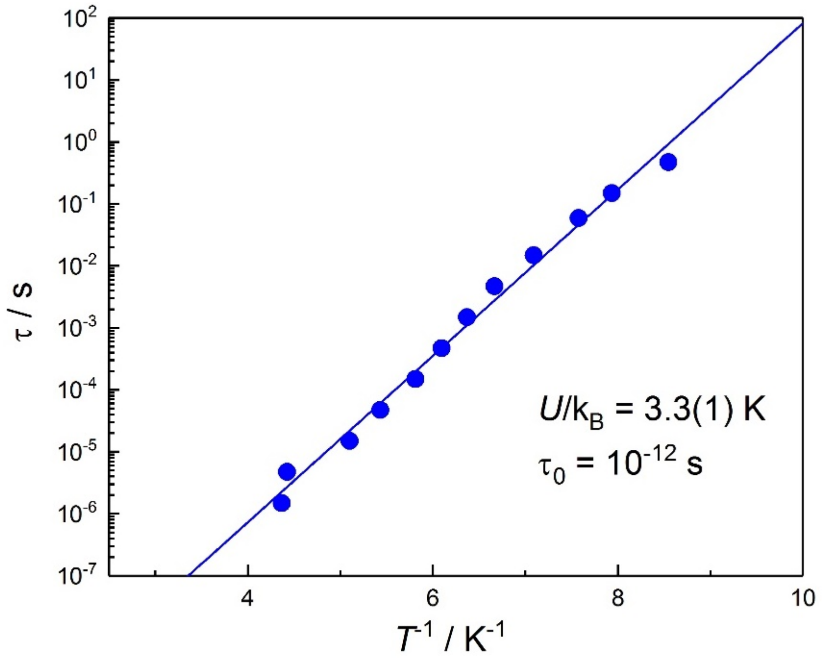


**Supplementary Figure 9** Arrhenius plot of the spin-lattice relaxation time (logarithmic scale), estimated from χ" vs $T$ maxima (dots). The solid line is a least-squares fit of an Arrhenius law $\tau = \tau_0 \exp(U/k_BT)$ to these data.

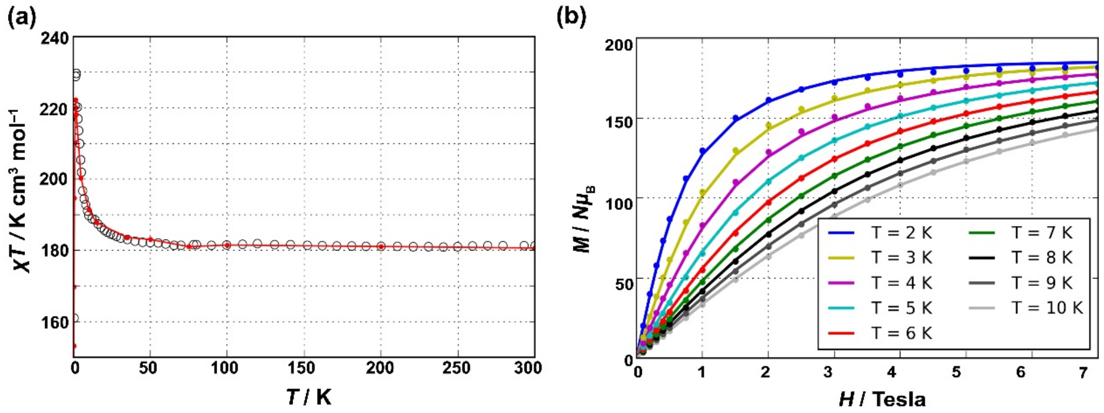


**Supplementary Figure 10** (**a**) $\chi T$ versus $T$. The experimental data are shown as circles, and the solid curve was computed using the anisotropic model with $J_1/k_B = -0.0225$ K, $J_2/k_B = -0.0113$ K, $J_3/k_B = -0.225$ K, and $D_{Ni}/k_B = 2.5$ K (best fit). (**b**) Magnetization versus field for several fixed values of temperature using the anisotropic model with the same best-fit parameters that were used in (a). The experimental data are shown as dots and theory data shown as solid curve.

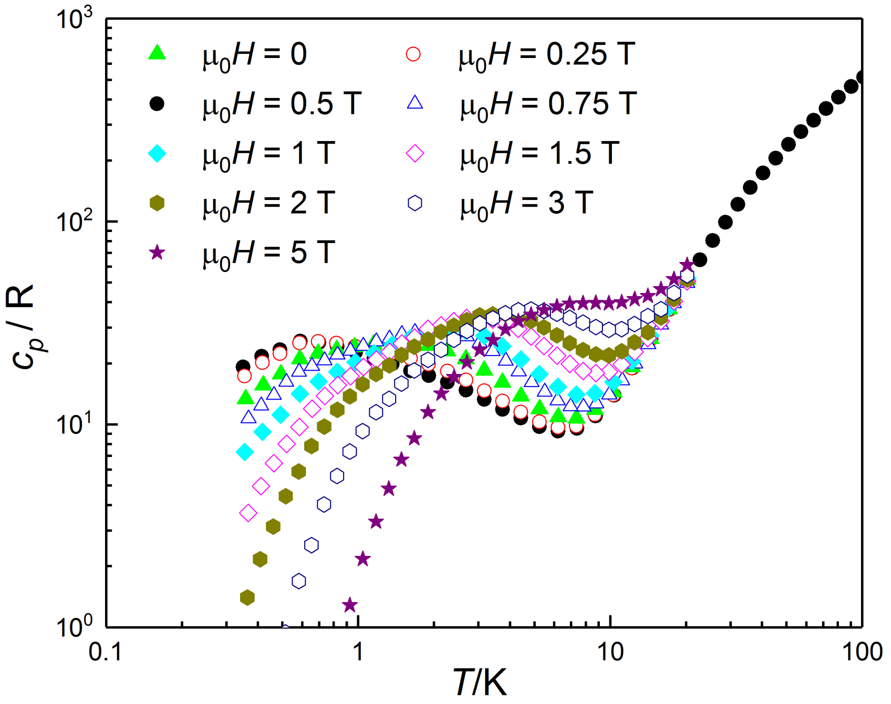


**Supplementary Figure 11** Specific heat of **1** measured at different magnetic fields as a function of temperature.

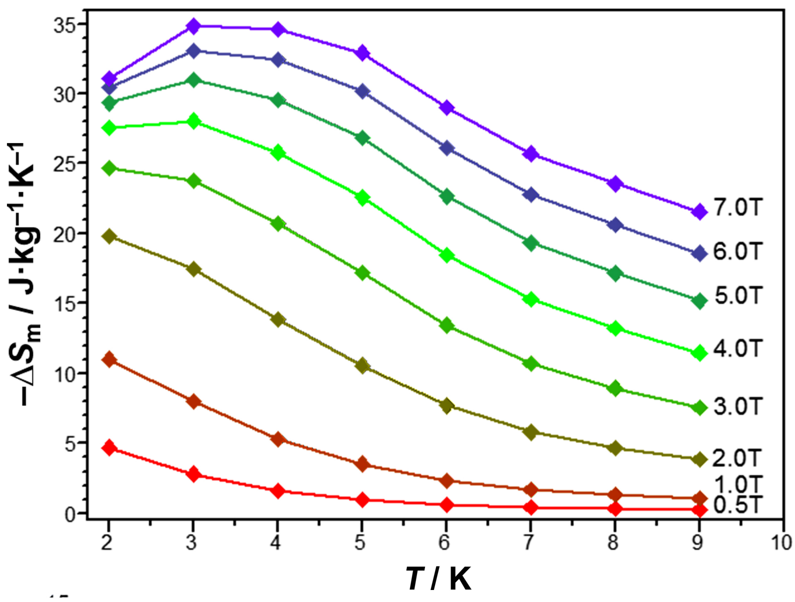


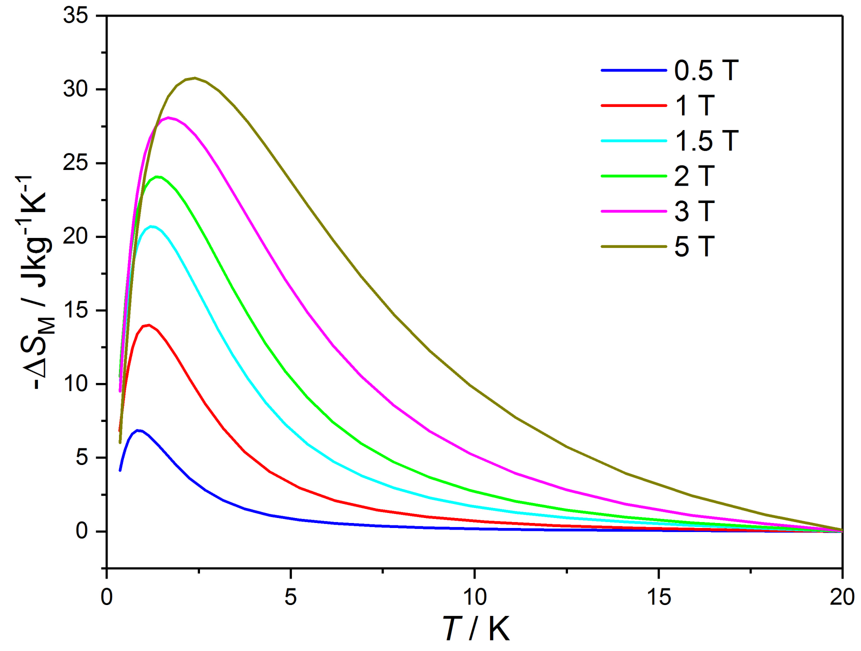


**Supplementary Figure 12** The plots of $-\Delta S_M$ for compound **1**. Top, data obtained from magnetization data. Bottom, data obtained from heat capacity data.

**Supplementary Table 1** Crystal Data and Structure Refinement for compound **1**.

| | |
|---|---|
| Compound | $\{Ni_{21}Gd_{20}\}$ (**1**) |
| Empirical formula | $C_{200}H_{362}N_{31}Ni_{21}Br_5Gd_{20}O_{236}$ |
| Formula weight | 11754.64 g/mol |
| Crystal system, space group | Hexagonal, $P6(3)/m$ |
| Unit cell dimensions[a] | $a$ = 21.659(2) Å, $\alpha$ = 90 deg. |
| | $c$ = 34.490(7) Å, $\gamma$ = 120 deg. |
| Volume | 14012(4) $Å^3$ |
| $Z$ | 2 |
| $D_x$ | 2.786 $Mg/m^3$ |
| Absorption coefficient | 6.89 $mm^{-1}$ |
| $F(000)$ | 11420 |
| Crystal size | 0.16 x 0.14 x 0.13 mm |
| Theta range for data collection | 1.09 to 25.00 deg. |
| Reflections collected / unique | 77461 / 8193 [$R$(int) = 0.0520] |
| Data / restraints / parameters | 8193 /2084 / 739 |
| Goodness-of-fit on $F^2$[b] | 0.99 |
| Final $R$ indices [$I$>2sigma(I)][c] | $R_1$ = 0.066, $wR_2$ = 0.175 |

[a] The $a$ and $c$ values were given by using a rhombohedral representation of the hexagonal space group.
[b] $GOF = [\Sigma w(F_o^2 - F_c^2)^2/(n_{obc} - n_{param})]^{1/2}$
[c] $R_1 = ||F_o|-|F_c||/\Sigma|F_o|$, $wR_2 = [\Sigma w(F_o^2 - F_c^2)^2/\Sigma w(F_o^2)^2]^{1/2}$

**Supplementary Table 2** Selected Bond Lengths (Å) and Bond Angles (°) for Compound **1**.

| The Selected Bond Lengths (Å) for Compound **1**. | | | |
|---|---|---|---|
| Ni1—O2 | 2.032 (11) | Ni1—O3 | 2.038 (12) |
| Ni1—O1 | 2.036 (12) | Ni1—O5 | 2.039 (11) |
| Ni1—O4 | 2.060 (12) | Ni2—O9 | 2.010 (11) |
| Ni2—O12 | 2.017 (12) | Ni2—O10 | 2.048 (12) |
| Ni2—O15 | 2.072 (13) | Ni2—O11 | 2.088 (12) |
| Ni3—O23 | 2.022 (13) | Ni3—O21 | 2.044 (12) |
| Ni3—O26 | 2.048 (13) | Ni3—O22 | 2.088 (12) |
| Ni3—O27 | 2.103 (15) | Ni4—O29 | 1.987 (16) |
| Ni4—O30 | 2.058 (12) | Ni4—O30$^{i}$ | 2.058 (12) |
| Ni4—O25$^{i}$ | 2.061 (12) | Ni4—O25 | 2.061 (12) |
| Ni1—N1 | 2.076 (14) | Ni2—N2 | 2.106 (15) |
| Ni3—N5 | 2.092 (14) | Ni4—N6 | 2.082 (19) |
| Gd1—O6 | 2.393 (10) | Gd1—O6$^{iii}$ | 2.393 (10) |
| Gd1—O6$^{ii}$ | 2.393 (10) | Gd1—O5$^{ii}$ | 2.435 (10) |
| Gd1—O5$^{iii}$ | 2.435 (10) | Gd1—O5 | 2.435 (10) |
| Gd1—O2$^{ii}$ | 2.453 (11) | Gd1—O2 | 2.453 (11) |
| Gd1—O2$^{iii}$ | 2.453 (11) | Gd2—O5$^{ii}$ | 2.352 (11) |
| Gd2—O9 | 2.372 (11) | Gd2—O13 | 2.374 (12) |
| Gd2—O6 | 2.470 (10) | Gd2—O7 | 2.475 (11) |
| Gd2—O10 | 2.505 (11) | Gd2—O16 | 2.528 (12) |
| Gd2—O8 | 2.560 (10) | Gd2—O1$^{ii}$ | 2.694 (11) |
| Gd3—O7 | 2.288 (10) | Gd3—O18 | 2.345 (14) |
| Gd3—O22 | 2.364 (12) | Gd3—O21 | 2.384 (12) |
| Gd3—O17 | 2.406 (13) | Gd3—O20 | 2.414 (13) |
| Gd3—O19 | 2.462 (12) | Gd3—O16 | 2.507 (12) |
| Gd3—O31 | 2.630 (10) | Gd4—O28 | 2.289 (8) |
| Gd4—O29 | 2.408 (9) | Gd4—O9$^{ii}$ | 2.443 (11) |
| Gd4—O21 | 2.461 (12) | Gd4—O30 | 2.505 (11) |
| Gd4—O11$^{ii}$ | 2.531 (12) | Gd4—O19 | 2.528 (12) |
| Gd4—O23 | 2.546 (11) | Gd4—O8$^{ii}$ | 2.715 (10) |
| The Selected Bond Angles (°) for Compound **1**. | | | |
| Gd2–O8–Gd4$^{iii}$ | 104.7(3) | Gd3–O16–Gd2 | 105.4(4) |
| Gd4–O29–Gd4$^{i}$ | 107.6(6) | Gd3–O19–Gd4 | 108.1(5) |
| Gd1–O6–Gd2 | 108.6(4) | Gd2$^{iii}$–O5–Gd1 | 111.2(4) |
| Gd3–O21–Gd4 | 113.0(5) | Gd3–O7–Gd2 | 114.5(4) |
| Gd4$^{i}$–O28–Gd4 | 116.2(6) | Gd2–O9–Gd4$^{iii}$ | 120.3(4) |
| Average Gd–O–Gd angle | | 110.96 | |

Symmetry codes: (i) x, y, −z+1/2; (ii) −x+y+1, −x+1, z; (iii) −y+1, x−y, z.

**Supplementary Table 3** $-\Delta S_M$ ( > 30 J·kg$^{-1}$·K$^{-1}$ ) for reported Gd-based polymetallic clusters.

| **Complexes** | $-\Delta S_M$ (J·kg$^{-1}$·K$^{-1}$) | ***T*** **(K)** | $\Delta H$ **(T)** | ***Ref*** |
|---|---|---|---|---|
| $\{Gd^{III}_{104}\}$ | 46.9 | 2.0 | 7 | 1 |
| $\{Gd^{III}_{24}\}$ | 46.1 | 2.5 | 7 | 2 |
| $\{Gd^{III}_{48}\}$ | 43.6 | 1.8 | 7 | 3 |
| $\{Ni^{II}_{64}Gd^{III}_{96}\}$ | 42.8 | 3.0 | 7 | 4 |
| $\{Co^{II}_{9}Co^{III}Gd^{III}_{42}\}$ | 41.3 | 2.0 | 7 | 5 |
| $\{Gd^{III}_{2}\}$ | 40.6 | 1.8 | 7 | 6 |
| $\{Ni^{II}_{10}Gd^{III}_{42}\}$ | 38.2 | 2.0 | 7 | 5 |
| $\{Gd^{III}_{38}\}$ | 37.9 | 1.8 | 7 | 3 |
| $\{Ni^{II}_{12}Gd^{III}_{36}\}$ | 36.3 | 3.0 | 7 | 7 |
| $\{Gd^{III}_{12}Mo_{4}\}$ | 35.3 | 3.0 | 7 | 8 |
| $\{Ni^{II}_{21}Gd^{III}_{20}\}$ (**1**) | 34.8 | 3.0 | 7 | **This work** |
| $\{Ni^{II}_{2}Gd^{III}_{2}\}$ | 34.4 | 4.5 | 7 | 9 |
| $\{Mn^{II}_{4}Gd^{III}_{6}\}$ | 33.7 | 3.0 | 7 | 10 |
| $\{Co^{II}_{6}Gd^{III}_{8}\}$ | 33.0 | 4.0 | 14 | 11 |
| $\{Co^{II}_{4}Gd^{III}_{10}\}$ | 32.6 | 2.0 | 7 | 12 |
| $\{Ni^{II}_{6}Gd^{III}_{6}\}$ | 32.0 | 3.0 | 7 | 13 |
| $\{Cu^{II}_{5}Gd^{III}_{4}\}$ | 31 | 3.0 | 9 | 14 |

**Supplementary Table 4** Comparison of the magnetisation values of 3d-4f / 4f clusters.

| Compounds | Magnetisation[a] ($N\mu_B$) | $XT$ value at 2K ($cm^3 \cdot K \cdot mol^{-1}$) | $XT$ value at RT[b] ($cm^3 \cdot K \cdot mol^{-1}$) | $\theta$ value (K) | Magnetic exchange | *Ref* |
|---|---|---|---|---|---|---|
| $\{Ni^{II}_{64}Gd^{III}_{96}\}$ | 779.5 | 625.0 | 811.2 | −1.15 (50-300K) | AFM and FM | 4 |
| $\{Gd^{III}_{104}\}$ | 708.4 | 278.48 | 804.26 | −4.11 (2-300K) | AFM | 1 |
| $\{Co^{II}_{9}Co^{III}Gd^{III}_{42}\}$ | 341.8 | ~175 | 402.26 | −4.27 (50-300K) | AFM | 5 |
| $\{Gd^{III}_{48}\}$ | 335.5 | 126.05 (1.8) | 376.60 | −3.57 (1.8-300K) | AFM | 3 |
| $\{Ni^{II}_{10}Gd^{III}_{42}\}$ | 298.4 | ~160 | 342.62 | −3.13 (50-300K) | AFM | 5 |
| $\{Gd^{III}_{38}\}$ | 265.9 | 142.99 (1.8K) | 299.06 | −2.99 (1.8-300K) | AFM | 3 |
| $\{Ni^{II}_{12}Gd^{III}_{36}\}$ | 225.7 | 113.36 | 290.0 | −3.95 (2-300K) | AFM | 7 |
| $\{Gd^{III}_{24}\}$ | 240.0 | 167.11 | 188.89 | -0.16 (30-300K) | AFM | 2 |
| $\{Co^{II}_{16}Gd^{III}_{24}\}$ | 185.4 | ~170 | 245.3 | $\theta < 0$ | AFM | 15 |
| **$\{Ni_{21}Gd_{20}\}$** | **182.0** | **227.7** | **179.7** | **+0.61 (100-300K)** | **FM** | **This Work** |
| $\{Cu^{II}_{36}Gd^{III}_{24}\}$ | 179.0 | ~108 | 203.8 | $\theta < 0$ | AFM | 16 |
| $\{Zn^{II}_{6}Gd^{III}_{24}\}$ | 154.8 | 85.0 | 177.6 | −1.91 (2-300K) | AFM | 17 |

[a.] Measured at 2 K and 7 T unless $\{Gd^{III}_{48}\}$, $\{Gd^{III}_{38}\}$ and $\{Cu^{II}_{36}Gd^{III}_{24}\}$ (at 1.8K and 7 T).

[b.] Room temperature.

## Supplementary References


1. Peng, J.-B. *et al*. Beauty, symmetry, and magnetocaloric effect−four-shell keplerates with 104 lanthanide atoms. *J. Am. Chem. Soc.* **136**, 17938 – 17941 (2014).
2. Chang, L.-X., Xiong, G., Wang, L., Cheng, P. & Zhao, B. A 24-Gd nanocapsule with a large magnetocaloric effect. *Chem. Commun.* **49**, 1055 – 1057 (2013).
3. Guo, F.-S. *et al.* Anion-templated assembly and magnetocaloric properties of a nanoscale $\{Gd_{38}\}$ cage versus a $\{Gd_{48}\}$ barrel. *Chem. Eur. J.* **19**, 14876 – 14885 (2013).
4. Chen, W.-P. *et al.* A mixed-ligand approach for a gigantic and hollow heterometallic cage $\{Ni_{64}RE_{96}\}$ for gas separation and magnetic cooling applications. *Angew. Chem. Int. Ed.* **55**, 9375 – 9379 (2016).
5. Peng, J.-B. *et al.* High-nuclearity 3d-4f clusters as enhanced magnetic coolers and molecular magnets. *J. Am. Chem. Soc.* **134**, 3314 – 3317 (2012).
6. Evangelisti, M. *et al.* Cryogenic magnetocaloric effect in a ferromagnetic molecular dimer. *Angew. Chem. Int. Ed.* **50**, 6606 – 6609 (2011).
7. Peng, J.-B. *et al.* A 48-metal cluster exhibiting a large magnetocaloric effect. *Angew. Chem. Int. Ed.* **50**, 10649 – 10652 (2011).
8. Zheng, Y. *et al.* Molybdate templated assembly of $Ln_{12}Mo_4$-type clusters (Ln = Sm, Eu, Gd) containing a truncated tetrahedron core. *Chem. Commun.* **49**, 36 – 38 (2013).
9. Wang, P., Shannigrahi, S., Yakovlev, N. L. & Andy Hor, T. S. Facile self-assembly of intermetallic $[Ni_2Gd_2]$ cubane aggregate for magnetic refrigeration. *Chem. Asian J.* **8**, 2943 – 2946 (2013).
10. Zheng, Y.-Z., Pineda, E. M., Helliwell, M. & Winpenny, R. E. P. $Mn^{II}$-$Gd^{III}$ phosphonate cages with a large magnetocaloric effect. *Chem. Eur. J.* **18**, 4161 – 4165 (2012).
11. Zheng, Y.-Z., Evangelisti, M., Tuna, F. & Winpenny, R. E. P. Co-Ln mixed-metal phosphonate grids and cages as molecular magnetic refrigerants. *J. Am. Chem. Soc.* **134**, 1057 – 1065 (2012).
12. Pineda, E. M. *et al.* Molecular amino-phosphonate cobalt–lanthanide clusters. *Chem. Commun.* **49**, 3522 – 3524 (2013).
13. Pineda, E.M., Tuna, F., Zheng, Y.-Z., Winpenny, R. E. P. & McInnes, E. J. *Inorg. Chem.* **52**, 13702 – 13707 (2013).
14. Langley, S. K. *et al.* Molecular coolers: the case for $[Cu^{II}_5Gd^{III}_4]$. *Chem. Sci.* **2**, 1166 – 1169 (2011).
15. Zhang, Z.-M. *et al.* Wheel-shaped nanoscale 3d–4f $\{Co^{II}_{16}Ln^{III}_{24}\}$ clusters (Ln = Dy and Gd). *Chem. Commun.* **49**, 8081 – 8083 (2013).
16. Leng, J.-D., Liu, J.-L. & Tong, M.-L. Unique nanoscale $\{Cu^{II}_{36}Ln^{III}_{24}\}$ (Ln = Dy and Gd) metallo-rings. *Chem. Commun.* **48**, 5286 – 5288 (2012).
17. Zhang, L. *et al.* Nanoscale $\{Ln^{III}_{24}Zn^{II}_6\}$ triangular metalloring with magnetic refrigerant, slow magnetic relaxation, and fluorescent properties. *Inorg. Chem.* **54**, 11535 – 11541 (2015).
